# One year monitoring of retinal morphologic and functional changes in traumatic optic neuropathy patients

**DOI:** 10.1186/s12886-024-03404-x

**Published:** 2024-03-25

**Authors:** Myungjin Kim, Helen Lew

**Affiliations:** Department of Ophthalmology, CHA Bundang Medical Center, CHA University, Seongnam, Republic of Korea

**Keywords:** Ganglion cell, Inner plexiform layer, Nerve fiber layer, Neuropathy, Optic nerve, Retina, Trauma

## Abstract

**Background:**

To analyze the morphologic and functional change in traumatic optic neuropathy (TON) divided by the mechanism of optic nerve injury.

**Methods:**

A retrospective analysis of 58 patients who were diagnosed as monocular TON from February 2015 to August 2021 was conducted at in CHA Bundang Medical Center in Seongnam, South Korea. The patients visited the clinic of the department of ophthalmology for more than 6 months and at least 4 times during this period.

**Results:**

44 patients were classified as blunt TON patients, and 14 patients were surgical TON patients. The visual acuity showed significant decrease in traumatic eyes at the first visit after injury compared to fellow eyes and maintained the injured status during the 1-year follow-up period in blunt TON. In surgical TON, the visual acuity slightly improved during 1 month follow-up period. RNFL thickness tended to be decreased at 1 month after first visit blunt TON patients, which was earlier than surgical TON patients. GCIPL thickness showed earlier decreased than RNFL thickness in both blunt and surgical TON patients.

**Conclusions:**

In both blunt and surgical TON eyes, there was a notable thinning in both RNFL and GCIPL, with particularly remarkable reduction in GCIPL in early phase. Therefore, analyzing each retinal layer thickness using OCT in conjunction with assessing visual function would be necessary. This combined approach is not only crucial for understanding clinical courses of each TON, but also predicting the morphological and functional deteriorations in TON.

## Background

Optic nerve damage usually causes secondary edema in parts of retinal ganglion cell (RGC) axons, resulting in RGC degeneration [1–3]. These degenerative changes lead to morphological changes in the retina and significant thinning of retinal layers. Previous studies have investigated the functional and morphological changes in the retina following optic nerve injury in patients with various optic nerve disorders, such as glaucoma, ischemic optic neuropathy, and optic neuritis [4–7].

Traumatic optic neuropathy (TON) occurs as a result of optic nerve injury following trauma, leading to acute axonal loss and severe visual impairment. TON is usually categorized into direct and indirect TON based on the mechanism of injury. Direct TON is typically caused by penetration or compression of an object into the orbit, whereas indirect TON results from the impact energy of a head contusion [1]. Recent clinical investigations have utilized optical coherence tomography (OCT) to study changes in the thickness of the retinal nerve fiber layer (RNFL) and ganglion cell-inner plexiform layer (GCIPL) in TON patients. Meier et al. observed RNFL thickness loss in 5 TON patients [8], while Cennamo et al. examined differences in RNFL and GCIPL thickness between the affected and unaffected eyes in 22 patients [9]. However, existing studies suffer from a limited sample size, and previous research did not categorize patients based on the mechanism of injury when evaluating morphological changes.

Therefore, we divided TON patients according to the injury mechanism and compared natural courses of each TON using functional and morphological parameters with larger samples. To the best of our knowledge, this is the first article to describe natural courses of TON divided by injury mechanism. This would provide a possible clue for the therapeutic window time for each TON.

## Methods

We retrospectively reviewed the medical records of 95 patients who were diagnosed as monocular TON in CHA Bundang Medical Center in Seongnam, South Korea, from February 2015 to August 2021. The study was approved by the Institutional Review board of CHA Bundang Medical Center (IRB file number: 2022-06-012). All study conduct adhered to the tenets of the Declaration of Helsinki. This study does not deal with experiments on humans and/or the use of human tissue samples.

The patients visited the clinic of the department of ophthalmology for more than 6 months and at least 4 times during this period. The diagnosis of TON was made when the decreased visual acuity or visual field were examined after head or periorbital trauma showing relative afferent pupillary defect without history of optic neuropathy. To address the reliability issue associated with poor visual acuity during visual field testing, we excluded patients with other ocular diseases that could lead to visual deterioration such as glaucoma, optic neuritis, media opacity, amblyopia and selected patients who could read more after refractive correction in the vision chart. Patients who could not read the vision chart were excluded initially.

Blunt TON was defined as TON resulted from traumatic accident, and surgical TON was defined as TON occurred after brain surgery. In surgical TON group, 11 patients underwent craniotomy for tumor removal near optic chiasm, and 3 patients underwent aneurysm clipping. At the first visit, ocular history was taken, and basic ophthalmic examination such as best-corrected visual acuity (BCVA), intraocular pressure measurement, slit lamp examination, fundus, OCT, and visual field examination were performed for both eyes. Then, the examinations were repeated at 1 month, 2 months, 6 months, and 1 year after the injury, except for the history check.

**Fig. 1:**
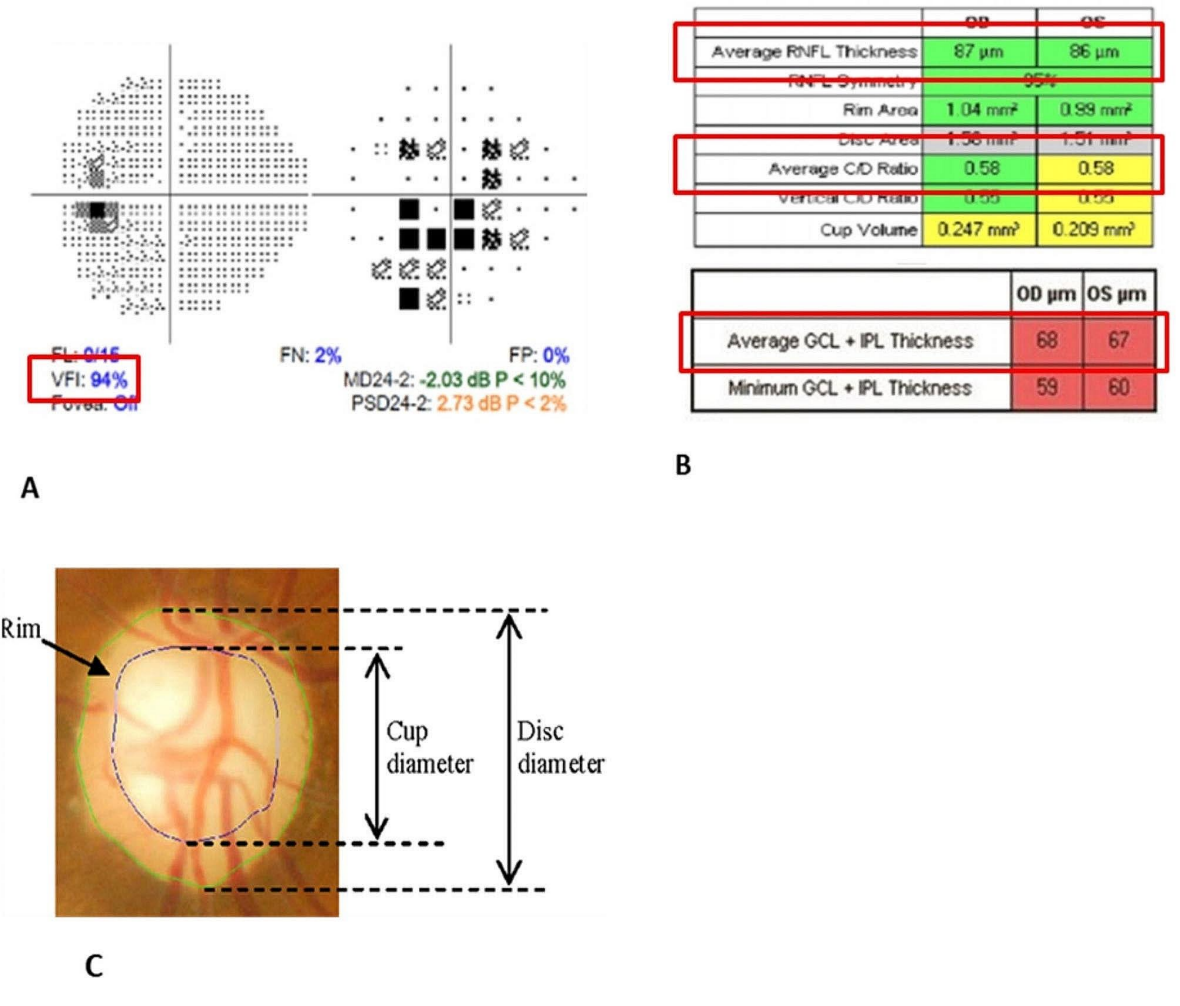
Measurement method of VFI, RNFL thickness, GCIPL thickness, and CD ratio. **(A)** VFI (Humphrey Field Analyzer (Carl Zeiss Ophthalmic Systems, Inc., Dublin, CA, USA)); **(B)** RNFL thickness and GCL thickness (Optical coherence tomography (Carl Zeiss Meditec, Dublin, CA, USA)); **(C)** CD ratio. *VFI* = visual field index; *RNFL* = retinal nerve fiber layer; *GCL* = ganglion cell layer

The visual acuity of all patients was converted into logMAR, and the difference in visual acuity between the patient’s traumatic eye and fellow eye was investigated. It was calculated by converting 3.0 logMAR for no light perception (NLP), 2.3 logMAR for light perception (LP), 2.0 logMAR for hand motion (HM), and 1.7 logMAR for count fingers(CF) [10]. Visual field test was performed using the 24 − 2 Swedish Interactive Thresholding Algorithm (SITA) standard of Humphrey Automated Perimetry (Carl Zeiss Meditec, Dublin, CA), and the visual field index (VFI) was evaluated (Fig. 1A). RNFL thickness and GCIPL thickness measurement were performed using Cirrus OCT (Carl Zeiss Meditec, Dublin, CA, USA). The Optic Disc Cube 200 × 200 scan was used to measure the average circumpapillary RNFL thickness and the average GCIPL thickness (Fig. 1B). Also, Cup/Disc ratio (CD ratio) was calculated with fundus photography (Fig. 1C).

All statistical analyses employed SPSS for Windows, version 27.0 (IBM Corp., Armonk, NY, USA). Mann Whitney U test were used to compare the clinical characteristics between the blunt TON and surgical TON groups. Significant probability (p-value) less than 0.05 was interpreted as statistically significant. To investigate the natural courses of variables of visual acuity, VFI, RNFL thickness, and GCIPL thickness according to the time, the statistical analysis plan included a technique for handling missing data across multiple time points. We utilized a regression analysis approach to impute missing values. In this process, we selected a linear regression model as our choice of algorithm. This model assumes a linear relationship between the variables X and Y. We then trained the regression model on the available data to capture this relationship. Subsequently, we used the trained model to predict missing values of visual acuity, VFI, RNFL thickness, and GCIPL thickness based on the known values of time points. These predicted values were then inserted into the dataset, effectively imputing the missing data points.

## Results

**Fig. 2:**
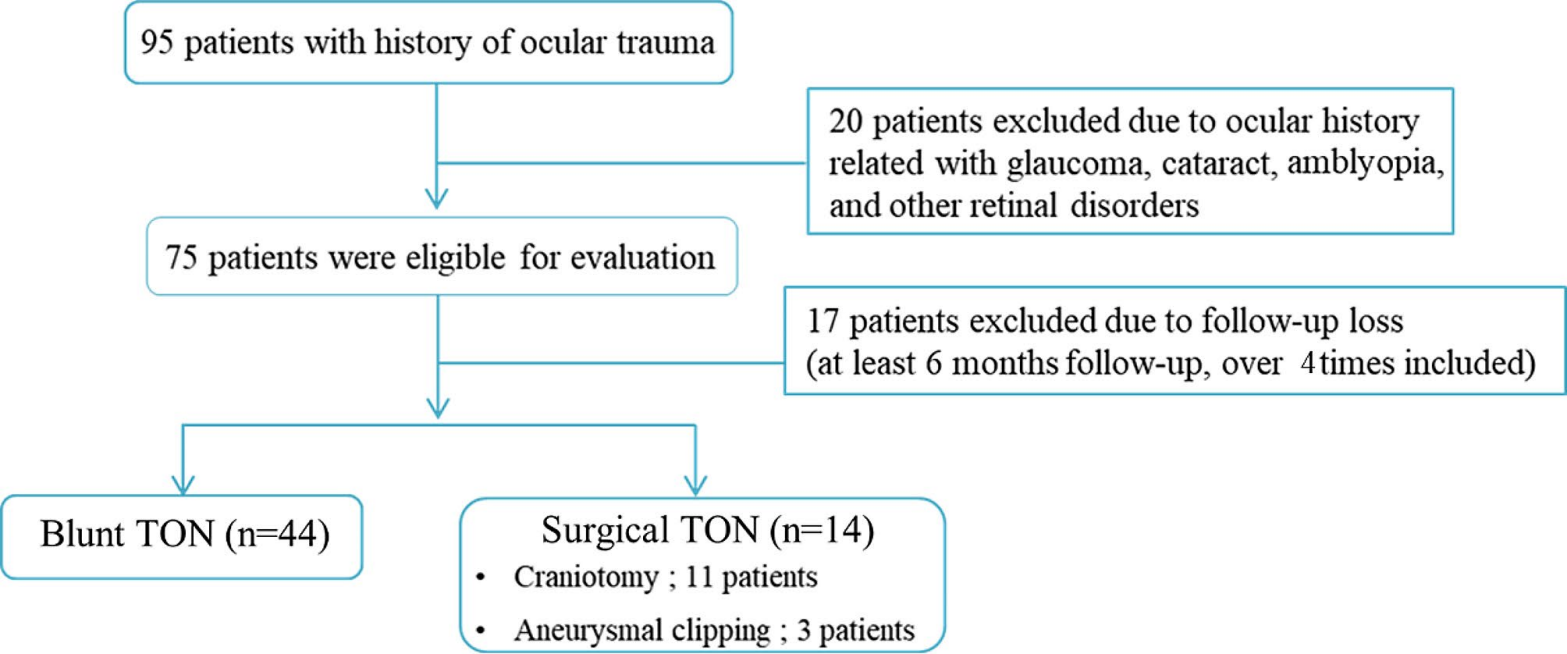
Study enrollment flow chart of patients with history of ocular trauma. Blunt TON was caused by blunt trauma and surgical TON was caused by brain surgical injury. *TON* = traumatic optic neuropathy

Overall, total 58 patients (45 males and 13 females) were included, 44 patients with blunt TON and 14 patients with surgical TON (Fig. 2). The average age of total patients was 47.7 ± 15.6 years at the time of diagnosis. The age between blunt TON patients and surgical TON patients differed (*p* < 0.001), with age of 33.4 ± 16.7 and 52.7 ± 15.6, respectively. There was no statistically significant difference in the sex and the history of steroid treatment (Table 1). The average follow-up period of patients was 242.2 ± 76.9 days, of which 18 patients continued follow-up for 1 year. Among them 11 patients were diagnosed as blunt TON and 7 patients were diagnosed as surgical TON.

**Table 1:** Clinical characteristics of total patients (*n* = 58) at first visit

	Injury mechanism			*p*-value
	Blunt (*n* = 44)	Surgical (*n* = 14)	Total (*n* = 58)	
Sex (male: female)	36: 8	9: 5	45: 13	0.141
Age (years)	33.4 ± 16.7 (33)	52.7 ± 15.6 (52)	47.4 ± 15.6 (42)	**< 0.001**
Steroid treatment (yes: no)	32: 12	12: 2	44: 14	0.134
**Traumatic eye**				
Visual acuity (LogMAR)	1.35 ± 1.05 (1.3)	1.38 ± 1.75 (1.3)	1.36 ± 0.29 (1.3)	0.769
VFI (%)	16.16 ± 5.51 (16)	13.81 ± 7.72 (14)	15.31 ± 6.37 (15)	0.232
RNFL thickness (µm)	96.44 ± 9.17 (95)	93.91 ± 10.92 (94)	95.32 ± 7.11 (94)	0.262
GCIPL thickness (µm)	68.44 ± 13.17 (69)	63.91 ± 12.92 (65)	65.22 ± 9.82 (67)	0.176
CD ratio	0.62 ± 0.11 (0.6)	0.57 ± 0.15 (0.6)	0.61 ± 0.11 (0.6)	0.723
**Fellow eye**				
Visual acuity (LogMAR)	0.25 ± 0.18 (0.22)	0.31 ± 0.28 (0.39)	0.29 ± 0.17 (0.30)	0.263
VFI (%)	88.35 ± 2.67 (89)	93.38 ± 4.87 (91)	90.33 ± 2.57 (90)	0.551
RNFL thickness (µm)	99.33 ± 6.07 (98)	95.78 ± 10.58 (96)	97.82 ± 7.48 (97)	0.701
GCIPL thickness (µm)	79.32 ± 4.07 (79)	75.08 ± 4.58 (75)	77.11 ± 2.44 (78)	0.064
CD ratio	0.53 ± 0.08 (0.5)	0.58 ± 0.13 (0.5)	0.56 ± 0.07 (0.5)	0.938

Values are presented as mean ± standard (median) deviation unless otherwise indicated. Comparative analyses were performed using Mann Whitney U test. Significant values with *P* < 0.05 are indicated in bold

**Fig. 3:**
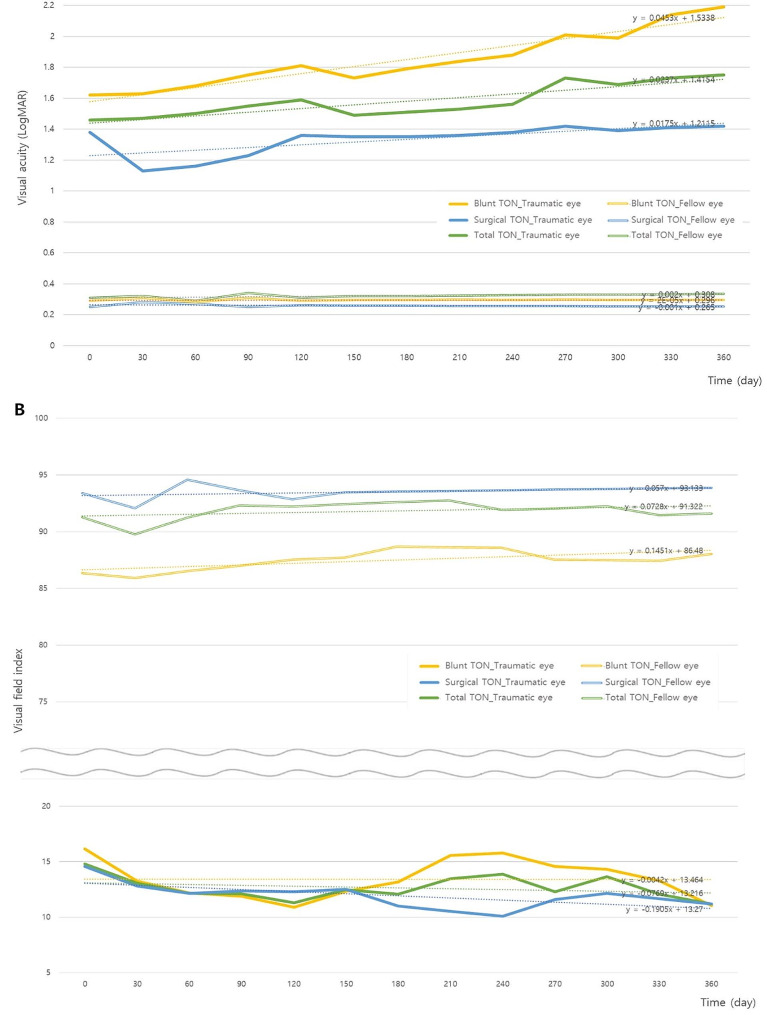
Natural courses of functional changes of visual acuity and VFI between traumatic eye and the fellow eye in TON patients. **(A)** Visual acuity; **(B)** VFI.

The visual acuity showed significant difference in traumatic eyes at the first visit after injury compared to fellow eyes and it was not improved during 1-year follow-up period in blunt TON. In contrast, in surgical TON patients, the visual acuity tended to be improved at month after the first visit. The average changes of visual acuity between the first and the last visit were 0.35 and 0.06 in blunt TON and surgical TON, respectively (Fig. 3A). The VFI also showed significant difference in traumatic eyes compared to fellow eyes at the first visit after injury, but it was not improved during 1-year follow-up period in both blunt and surgical TON. The average changes of VFI between the first and the last visit were − 5.10 and − 3.39 in blunt TON and surgical TON, respectively (Fig. 3B).

**Fig. 4:**
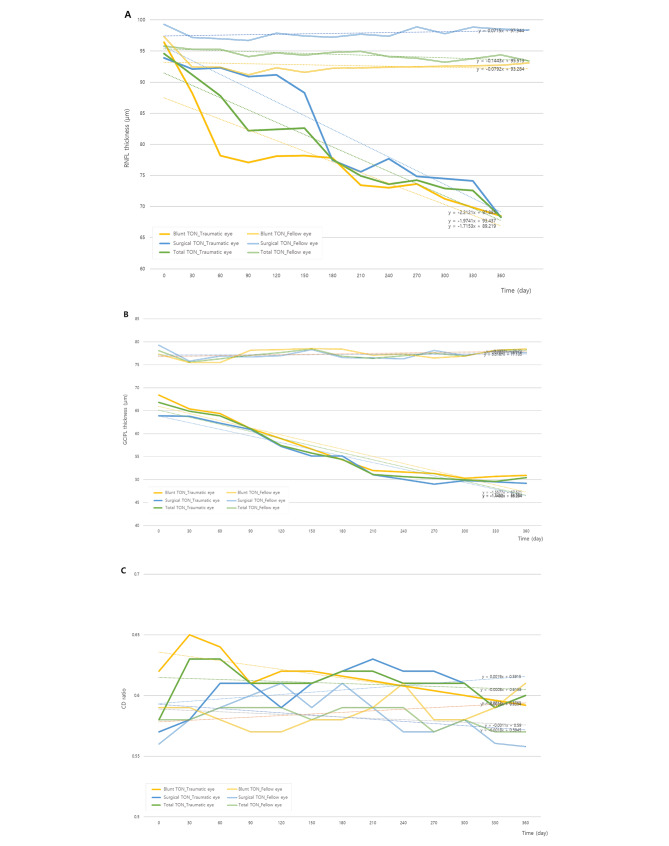
Natural courses of morphologic changes of RNFL thickness, GCIPL thickness, and CD ratio between traumatic eye and the fellow eye in TON patients. **(A)** RNFL thickness change; **(B)** GCIPL thickness change; **(C)** CD ratio

The circumpapillary RNFL thickness was 96.4 ± 9.2 μm in traumatic eye and 99.3 ± 6.1 μm in fellow eye in blunt TON patients at the first visit after trauma. In surgical TON patients, the circumpapillary RNFL thickness was 93.9 ± 10.9 μm in traumatic eye and 95.8 ± 10.5 μm in fellow eye at the first visit after trauma (Table 1). RNFL thickness started to decrease at 1 month after first visit in blunt TON, which was earlier than in surgical TON which showed decrease started from 6 month after the injury. The average changes of RNFL thickness between the first and the last visit were − 23.82 and − 19.72 in blunt TON and surgical TON, respectively (Fig. 4A). The GCIPL thickness was 68.4 ± 13.2 μm in traumatic eye and 79.3 ± 4.1 μm in fellow eye in blunt TON patients at the first visit after trauma. In surgical TON patients, the GCIPL thickness was 63.9 ± 13.9 μm in traumatic eye and 75.1 ± 4.6 μm in fellow eye at the first visit after trauma (Table 1). GCIPL thickness started to decrease at 1 month after first visit in both blunt TON and surgical TON patients. The average changes of GCIPL thickness between the first and the last visit were − 17.52 and − 14.79 in blunt TON and surgical TON, respectively (Fig. 4B). The CD ratio was 0.63 ± 0.11 in traumatic eye and 0.53 ± 0.08 in fellow eye in blunt TON patients at the first visit after trauma. There was no statistically significant difference in CD ratio between trauma eye and fellow eye in both blunt TON and surgical TON patients from the first visit to 1 year after first visit (Fig. 4C).

## Discussion

TON is a type of optic nerve injury that occurs as a result of trauma. RGCs degeneration due to axonal damage may also occur in patients with TON. In addition, as TON causes loss of RGCs and their axons, the optic nerve damage due to TON can affect the RNFL and GCIPL. Given that RGC synapses are located in the inner reticular layer, alterations in this layer are expected after optic nerve injury [11]. Timely diagnosis and appropriate management of TON rely on a comprehensive understanding of its characteristics and pathophysiology, which is important for determining diagnosis and treatment. Although TON may not exhibit abnormal retinal findings during early phases, changes over time due to nerve damage can be monitored using OCT [12, 13].

Some studies have reported retinal layer thickness to investigate the decrease in retinal activity after optic nerve injury. They reported up to a 40% reduction in the thickness of the RNFL in five TON patients [8]. Others investigated the distinct differences in RNFL and GCIPL thickness between the traumatic eyes and fellow eyes in TON patients whose onset of blunt trauma was valuable from one month to 55 years [9]. However, most studies had small sample sizes and did not investigate longitudinal changes in retinal morphological features and visual function in TON patients. In addition, most studies have not divided TON according to the mechanism of damage. Therefore, this study was conducted with a larger sample size and compared the two types of blunt and surgical injuries to evaluate the clinical course of TON patients.

Functional markers such as visual acuity and VFI showed significant reductions in the traumatic eye compared with the fellow eye from the first post-injury visit and over a 1-year follow-up period in both indirect and direct TON patients. Regarding the morphological findings with RNFL and GCIPL thickness changes, RNFL thickness showed an early loss in blunt TON compared to the surgical TON. On the other hand, GCIPL thickness was observed to decrease simultaneously in both blunt and surgical TON at 1 month after the first visit. Thickness change in GCIPL might be a more sensitive parameter to predict retinal adversity in TON despite the cause of optic nerve damage. It also suggests that RNFL thickness may be a useful marker to determine retinal affliction in blunt TON.

Ischemia probably plays a pivotal role in both the initial neuronal edema and the subsequent neuronal cell death and axonal atrophy seen in different stages of TON [14]. Ustymowicz. et al. documented a significant reduction in blood flow within the central retinal artery of affected eyes in TON patients [15]. Additionally, Shi et al. observed that changes in the parameters of the central retinal artery, which primarily supplies blood to the optic nerve and inner retina, were particularly pronounced among the vasculature parameters studied [16]. Furthermore, surgical TON may be more susceptible to ischemic conditions compared to blunt TON, potentially due to additional neurological stresses. These stresses include inflammatory mediator triggering macrophage and polymorphonuclear cell infiltration, resulting in the release of enzymes and free radicals that can further damage axons [17, 18].

The time of post-traumatic retinal morphological changes should be particularly noted in relation to the time of optic atrophy. Optic atrophy is a well-known consequence of TON and is characterized by a gradual pale and atrophic optic disc that occurs 3–5 weeks after trauma [19]. However, we detected a tendency of RNFL thickness to decrease and a marked reduction in GCIPL thickness in TON patients. This finding suggests that the morphological changes in GCIPL may precede the changes in RNFL in early TON and that it may occur before optic atrophy. This is an important observation as it suggests that GCIPL thickness may be a more sensitive parameter for predicting retinal injury in TON, regardless of the cause of optic nerve damage. Furthermore, RNFL thickness may be a useful marker for determining retinal damage in blunt TON. Overall, this finding highlights the importance of monitoring both RNFL and GCIPL thickness in TON patients to predict and distinguish the morphological destruction and function deterioration according to the types of TON.

Several limitations should be considered when interpreting the findings of this study. Firstly, the retrospective design of the study may have introduced inherent biases and limitations, such as selection bias and the inability to establish causality. Moreover, the relatively small sample size, encompassing only 18 patients followed up for 1 year, might restrict the generalizability of the findings. The lack of randomization and potential confounding variables among the blunt and surgical TON groups could also affect the comparability of the outcomes. Additionally, the handling of missing data using regression analysis for imputation may introduce potential biases and affect the robustness of the results.

Despite the aforementioned limitations, this study offers several strengths that contribute to the understanding of TON. The inclusion of both blunt and surgical TON patients, along with a comprehensive evaluation of retinal morphological features and visual function, provides valuable insights into the clinical course of TON patients. The longitudinal evaluation of retinal layer thickness and functional markers over a 1-year follow-up period supplements existing literature by capturing the dynamic changes in TON progression. Furthermore, the focus on distinct variances in retinal layer thickness between affected and unaffected eyes, as well as the comparison of blunt and surgical injuries, enhances our understanding of the morphological and functional changes associated with different mechanisms of optic nerve injury.

In conclusion, regardless of the limitation as a retrospective design, we could discover the significant thinning of RNFL and GCIPL in both blunt and surgical TON eyes, with remarkable reduction of GCIPL in early phase. Therefore, analyzing each retinal layer thickness using OCT as well as visual function would be helpful to understand clinical course of each TON and predict the morphological destruction and function deterioration in TON. Moreover, evaluating not only the RNFL thickness but also the GCIPL thickness in traumatic eyes could be useful to predict and distinguish the morphological destruction and function deterioration according the types of TON. Further studies with larger sample size and longer follow-up periods are warranted to validate the findings.

## Data Availability

The datasets used and/or analyzed during the current study available from the corresponding author on reasonable request.

## Abbreviations

BCVA: Best-Corrected Visual Acuity
CD ratio: Cup/Disc ratio
CF: Count Fingers
GCIPL: Ganglion Cell-Inner Plexiform Layer
HM: Hand Motion
LP: Light Perception
NLP: No Light Perception
OCT: Optical Coherence Tomography
RNFL: Retinal Nerve Fiber Layer
TON: Traumatic Optic Neuropathy
VFI: Visual Field Index
